# Automatic and efficient pneumothorax segmentation from CT images using EFA-Net with feature alignment function

**DOI:** 10.1038/s41598-023-42388-4

**Published:** 2023-09-15

**Authors:** Yinghao Liu, Pengchen Liang, Kaiyi Liang, Qing Chang

**Affiliations:** 1 School of Health Science and Engineering, University of Shanghai for Science and Technology, Shanghai, 200093 China; 2 Shanghai University of Medicine and Health Sciences, Shanghai, 200237 China; 3 Department of Surgery, Shanghai Key Laboratory of Gastric Neoplasms, Shanghai Institute of Digestive Surgery, Ruijin Hospital, Shanghai Jiao Tong University School of Medicine, Shanghai, 200025 China; 4 School of Microelectronics, Shanghai University, Shanghai, 201800 China; 5 Department of Radiology, Jiading District Central Hospital Affiliated Shanghai University of Medicine & Health Sciences, Key Laboratory of Shanghai Municipal Health Commission for Smart Image, Shanghai, 201800 China

**Keywords:** Biomedical engineering, Tomography

## Abstract

Pneumothorax is a condition involving a collapsed lung, which requires accurate segmentation of computed tomography (CT) images for effective clinical decision-making. Numerous convolutional neural network-based methods for medical image segmentation have been proposed, but they often struggle to balance model complexity with performance. To address this, we introduce the Efficient Feature Alignment Network (EFA-Net), a novel medical image segmentation network designed specifically for pneumothorax CT segmentation. EFA-Net uses EfficientNet as an encoder to extract features and a Feature Alignment (FA) module as a decoder to align features in both the spatial and channel dimensions. This design allows EFA-Net to achieve superior segmentation performance with reduced model complexity. In our dataset, our method outperforms various state-of-the-art methods in terms of accuracy and efficiency, achieving a Dice coefficient of 90.03%, an Intersection over Union (IOU) of 81.80%, and a sensitivity of 88.94%. Notably, EFA-Net has significantly lower FLOPs (1.549G) and parameters (0.432M), offering better robustness and facilitating easier deployment. Future work will explore the integration of downstream applications to enhance EFA-Net’s utility for clinicians and patients in real-world diagnostic scenarios. The source code of EFA-Net is available at: https://github.com/tianjiamutangchun/EFA-Net.

## Introduction

Pneumothorax, a medical condition marked by the abnormal presence of air within the pleural cavity, results in lung compression, dyspnea, cough, and potentially severe complications. This condition demonstrates a high recurrence rate, particularly in patients with chest injuries, where the incidence surpasses 30%^1^. The etiology of pneumothorax is complex and multifaceted, with contributing factors such as chest trauma, cough, smoking, exercise, and various lung disorders. Wakai et al.’s 2011 survey revealed that between 130,000 and 210,000 cases of pneumothorax occur annually in Western nations, including Europe and the United States, with an elevated recurrence rate, notably in males (35%)^2^. In the United States alone, approximately 7.4% of pneumothorax patients undergo delayed treatment as a consequence of missed or postponed diagnoses each year. This diagnostic difficulty stems from the appearance of pneumothorax as a dark area on computed tomography (CT) scans, which can easily overlap with chest scapulae and clavicles, and its elusive nature that complicates detection^3^. As such, expeditious pneumothorax screening and prompt clinical intervention are vital for affected individuals, emphasizing the importance of precise and efficient CT image segmentation for informed clinical decision-making.

**Figure 1:**
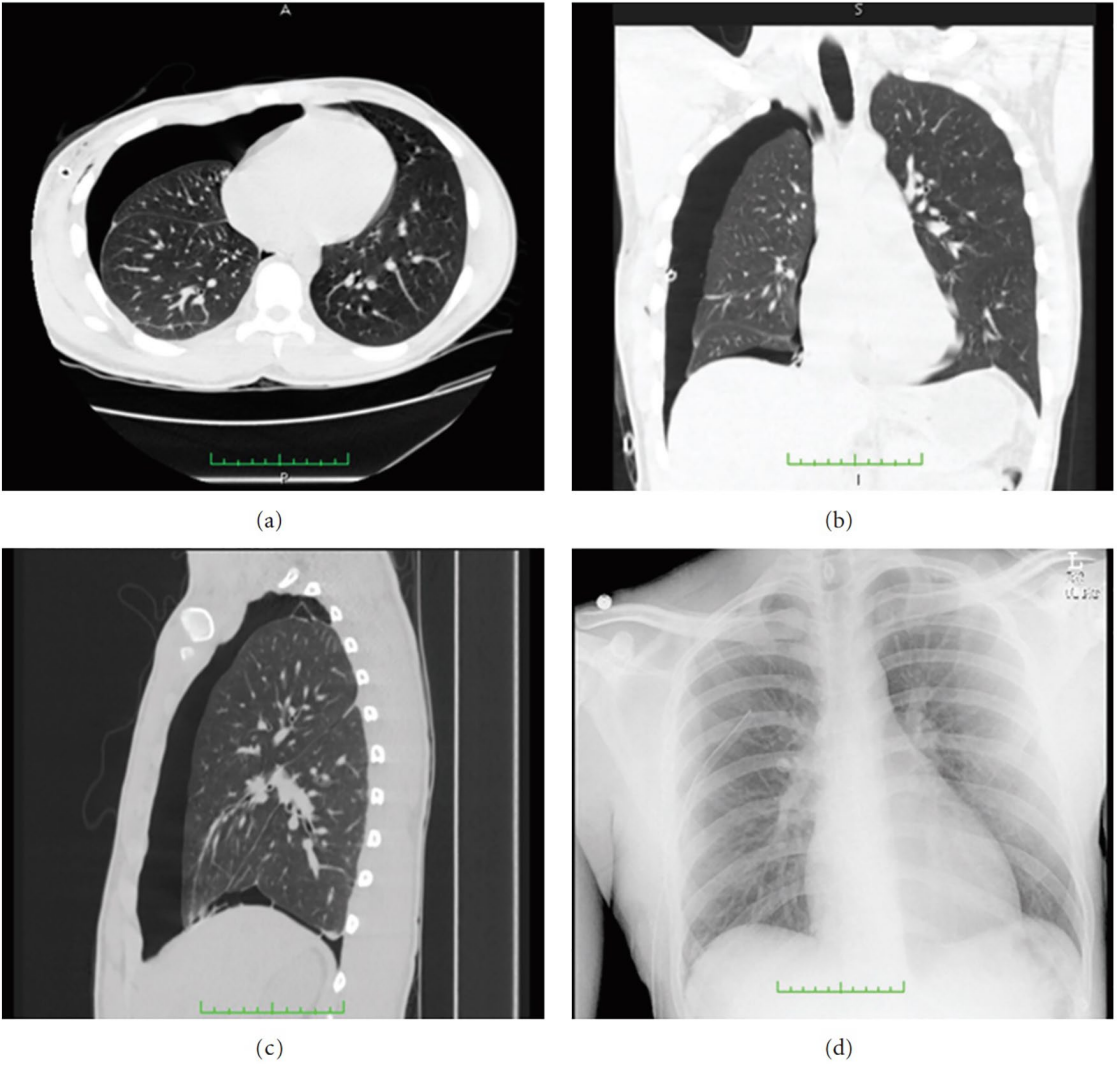
The CT images in transverse (**a**), coronal (**b**), and sagittal (**c**) views reveal a moderate to large pneumothorax in a patient, while the chest X-ray image (**d**) of the same patient taken two hours earlier lacks the level of detail provided by the CT images. The CT images are more effective in detecting and measuring the size of the pneumothorax.

Chest X-ray is a widely used diagnostic tool for rapid pneumothorax volume estimation. However, X-ray imaging presents limitations in pneumothorax detection and localization, particularly in cases involving pulmonary emphysema or obesity. Chest X-ray encounters three primary challenges: (1) imprecise and inconsistent volume estimates derived from a single image, (2) frequent misdiagnosis of small or localized pneumothorax, and (3) difficulty differentiating pneumothorax from similar lung diseases, such as bullae and emphysema. In comparison, computed tomography (CT) scans deliver more accurate lung anatomical data and provide clearer images of pneumothorax sites^3^, enabling differentiation between mild and moderate pneumothorax and conferring substantial advantages in diagnosis, as illustrated in Fig. 1.

The interpretation of CT images for pneumothorax detection is challenged by the pneumothorax area’s dark appearance, which can easily overlap with adjacent structures like the scapula and clavicle. This elusive and challenging-to-detect characteristic can result in misdiagnosis and delayed treatment^4^. Medical deep learning offers a solution to these challenges by facilitating precise, automatic pneumothorax segmentation on CT scans, thus reducing radiologists’ workload and ensuring accurate, timely diagnoses. The development of deep learning models capable of accurately detecting and segmenting pneumothorax areas is essential for decreasing the incidence of delayed treatment and enhancing patient outcomes. Nonetheless, the majority of deep learning-based pneumothorax segmentation research is centered on radiographs, with no open-source CT pneumothorax dataset accessible and a limited number of studies on chest CT pneumothorax^5^.

Deep learning (DL), particularly convolutional neural networks (CNNs)^6^, has made significant advancements in the medical imaging field, achieving remarkable success in various computer vision tasks such as image classification and segmentation. Neural networks have been effectively used to detect abnormal signals and segment lesion areas for clinical diagnosis. One efficient CNN technique involves treating image segmentation as semantic segmentation, assigning each image pixel a class label and providing a comprehensive image understanding^7^. The fully convolutional network (FCN) proposed by Long, Shelhamer, and Darrell is a semantic segmentation landmark and serves as the foundation for most modern methods. Ronneberger and Fischer proposed an FCN encoder-decoder network called U-Net, which has been successful in biomedical image segmentation. The U-Net architecture employs skip connections to achieve precise pixel-level localization, making it popular among researchers^8^.

Clinical practice requires medical imaging segmentation models to provide not only high-precision results and high-quality masks with high resolution but also fast processing speeds and low memory costs. The speed and memory efficiency of medical image segmentation models are critical factors for clinical applications, especially in real-time or near-real-time scenarios where quick and accurate diagnosis is necessary. Consequently, there is a growing demand for medical image segmentation models that balance accuracy, speed, and memory usage, which can be deployed on resource-limited hardware for point-of-care diagnosis or remote medical imaging applications. Achieving this balance between accuracy, speed, and memory usage remains a challenge for researchers and practitioners in the field of medical imaging segmentation.

Integrating high-level contextual information with low-level details is essential for semantic segmentation. To accomplish this, most existing segmentation models, such as DeepLab^9^, LinkNet^10^, and U-Net^8^, employ bilinear up-sampling and convolutions on feature maps at different scales before aligning them at a uniform resolution. However, bilinear up-sampling tends to blur the precise information encoded in these feature maps, and convolutions introduce additional computational overhead. These challenges are particularly acute in medical applications like pneumothorax segmentation, where the exact representation of intricate structures is vital. To address this, we introduce the Feature Alignment (FA) module into our model for pneumothorax segmentation. FA enables precise alignment without the blurring associated with bilinear up-sampling, minimizes computational complexity by avoiding unnecessary convolutions, and offers the flexibility to adapt to various coordinates and resolutions. These qualities make the FA module an efficient and precise solution for pneumothorax segmentation, effectively capturing the subtle feature differences required for accurate diagnosis, while significantly improving both segmentation accuracy and computational efficiency.

In this paper, we propose an EfficientNet-b5-based CNN model with a Feature Alignment Function (EFA-Net) for CT pneumothorax segmentation. Specifically, we use EfficientNet-b5 as the encoder^1^, leveraging its efficient convolutional neural network structure. EfficientNet is capable of extracting multiscale feature maps from input images. We employ the Feature Alignment function module as the decoder, a novel function that effectively and accurately aggregates features at different levels for semantic segmentation^11^. We construct EFA-Net by combining EfficientNet-b5 and the FA module, resulting in a general encoder-decoder structure akin to U-Net. Our experimental results demonstrate that our method outperforms six state-of-the-art approaches with lower Flops and parameters.

The structure of this paper is organized as follows: In “Related works” section, we present a comprehensive review of the existing literature on pneumothorax segmentation and the application of deep learning methodologies in medical image segmentation. In “Method” section details our proposed EFA-Net, including the EfficientNet-b5 encoder and the FA module as the decoder, and introduces the dataset used for evaluation. In “Experiments” section, we describe the experimental setup and the performance metrics utilized to evaluate the effectiveness of our method, as well as showcase the experimental results and comparisons with state-of-the-art techniques. Finally, “Conclusions” section concludes the paper by summarizing our contributions and highlighting prospective future research avenues in the realm of CT pneumothorax segmentation.

In summary, our key contributions in this paper are as follows:

- Utilization of authentic pneumothorax case data from clinical settings for our investigation, addressing the scarcity of studies focusing on CT pneumothorax segmentation.
- Proposal of an innovative CNN for CT pneumothorax segmentation, employing EfficientNet-b5 as the encoder and the FA module as the decoder.
- Evaluation of our method using a proprietary CT pneumothorax dataset, demonstrating superior Dice vs. IoU results with fewer parameters and FLOPs compared to six state-of-the-art approaches.

## Related works

Pneumothorax is a life-threatening condition characterized by the accumulation of air in the pleural space. Segmentation of pneumothorax is a critical task that assists in diagnosis. Most existing pneumothorax segmentation methods rely on chest X-ray images^5,12–18^, which are limited by factors such as low resolution, projection artifacts, and poor contrast between pneumothorax and normal lung tissues. These methods utilize texture features of traditional approaches^5,12–17,19,20^, semantic segmentation models^5,12–15,17^, or weakly supervised learning^13,21^. Hybrid approaches combining automated and manual segmentation techniques have also been developed for CT scans^20^, along with methods that employ machine learning for lung contour detection in 3D-CT scans^19^. The A-LugSeg method integrates automation and explainability for multi-site lung detection in chest X-ray images^22^. However, all of these methods may struggle to capture the subtle and complex boundaries of pneumothorax, particularly in cases of small or partial pneumothorax.

Traditional image processing methods^17^ employ image intensity and gradient features to discern subtle texture differences between pneumothorax and normal lung tissues but are hampered by low accuracy and smoothness due to limited data availability and variability.

Recently, deep learning methods have demonstrated improved performance in pneumothorax segmentation by employing pixel-level classification networks such as U-Net^5,12,15,17,20^, FC-DenseNet^14^, or DeepLabv3+^13^, and mUnet^23^. These methods assign a label to each pixel to indicate its association with pneumothorax. Although deep learning methods have shown promising results, they face limitations, including data scarcity and variability, which can result in overfitting and poor generalization performance. Furthermore, some deep learning methods rely on pixel-level classification, potentially hindering accurate capture of complex pneumothorax boundaries. Existing methods also fail to effectively exploit multi-level features. Traditional methods depend on texture features or image intensity and gradient features, while deep learning methods may sometimes emphasize low-level features, particularly in complex tasks like pneumothorax segmentation. This focus on low-level features can lead to segmentation errors when dealing with small or partial pneumothorax regions.

Our proposed method addresses these limitations by employing EfficientNet as an encoder and a Feature Alignment (FA) function as a decoder. Capitalizing on the powerful representation learning capabilities of EfficientNet and incorporating multi-level features through FA, our method achieves greater accuracy and is more lightweight, particularly for small or partial pneumothorax regions. Additionally, our method effectively handles data variability by learning to align features across different levels and scales, enhancing generalization performance and reducing overfitting, Higher accuracy with lower computation and parametric quantities can be obtained.

## Method

Our objective is to develop a model with the best segmentation performance and the lowest possible number of parameters, laying the groundwork for subsequent research. In this section, we will briefly introduce our dataset, discuss the encoder-decoder architecture for semantic segmentation, EfficientNet encoder and FA decoder, and then introduce the implementation details.

### dataset

The CT data obtained in this study were Nii suffix files, subsequently converted into DICOM files (Digital Imaging and Communications in Medicine) for use as training and test sets. DICOM is widely utilized in radiology, cardiovascular imaging, and diagnostic radiology equipment (X-ray, CT, MRI, ultrasound, etc.), with increasing applications in other medical fields, such as ophthalmology and dentistry.

All chest CT slices were sourced from Jiading District Central Hospital, affiliated with Shanghai University of Medicine and Health Sciences, Shanghai, China. The dataset includes 60 pneumothorax patients, randomly selected from routine clinical CT scans. Four radiologists performed pixel-level manual annotations of pneumothorax areas for axial slices using ITK-SNAP, which were subsequently reviewed by an experienced radiologist. Our dataset comprises 17,297 CT slices of size 512 × 512, with 12,535 slices containing pneumothorax areas. The dataset is divided into training, validation, and testing sets composed of 50, 4, and 6 pneumothorax patients respectively. The ethical part of this study was reviewed and approved by the Ethics Committee of Jiading District Central Hospital affiliated to Shanghai Health Medical College.

**Figure 2:**
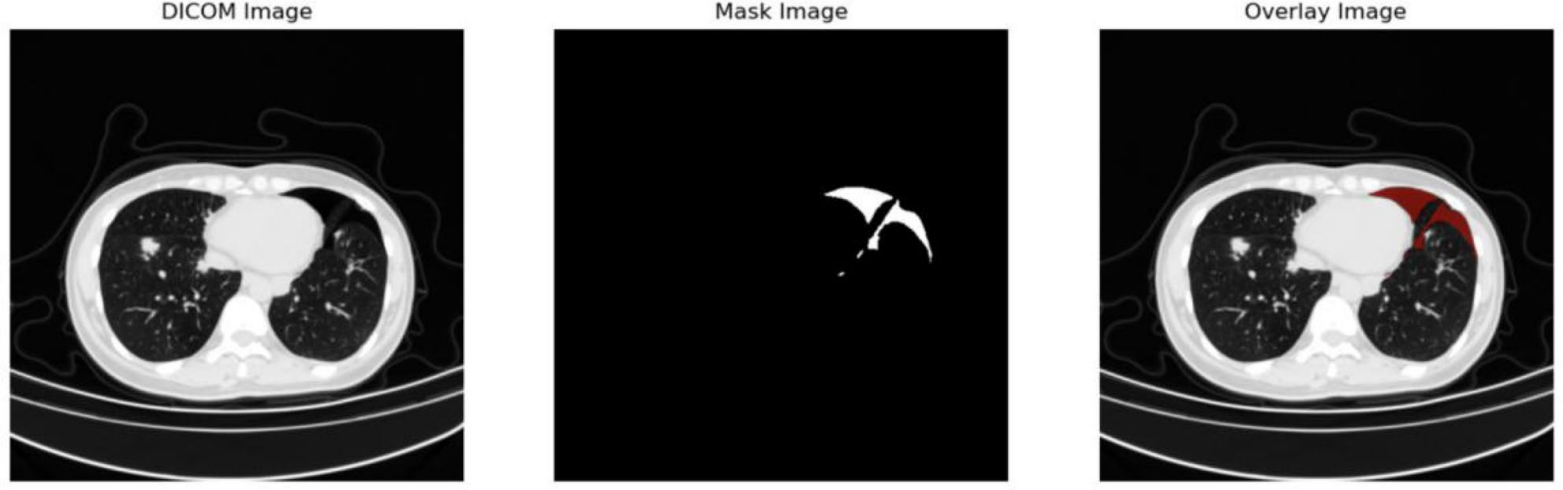
Examples of 3 different CT images (left), the masks (middle), and the CT images with masks (right).

Figure 2 presents CT images of pneumothorax disease, randomly selected from the dataset with physician-labeled masks, as well as image and mask.

### Encoder and decoder architecture

The encoder-decoder module is widely employed for image segmentation tasks. The encoder, a convolutional neural network (CNN), extracts feature from the original image. It progressively downsamples the image to capture high-level details while reducing the feature map resolution. State-of-the-art CNN architectures, such as U-Net^8^, Unet++^21^, EfficientNet^1^, mUnet^23^ among others, are typically used for this purpose. These architectures are designed to progressively reduce the input resolution of the image to obtain the final feature map in these classic models. And through the downsampling part of Decoder, the final feature map works transform feature maps to the same resolution for alignment, where bilinear upsampling blurs the precise information and convolutions can be inefficient.

### EfficientNet encoder

In the optimization of CNN-based networks, common approaches include increasing the network’s depth to obtain deeper and more complex feature maps or widening the network to achieve finer-grained features. However, both strategies encounter distinct challenges. Increasing depth may lead to vanishing gradients or training difficulties, while widening the network allows for rapid training but results in shallow depth due to the increased width, hindering the learning of deeper features. EfficientNet was introduced to simultaneously rationalize depth, width, and channel parameters, achieving the highest accuracy of 84.3% on ImageNet top-1 at the time and requiring only 1/8.4 of the parameter count then state-of-the-art models.

**Figure 3:**
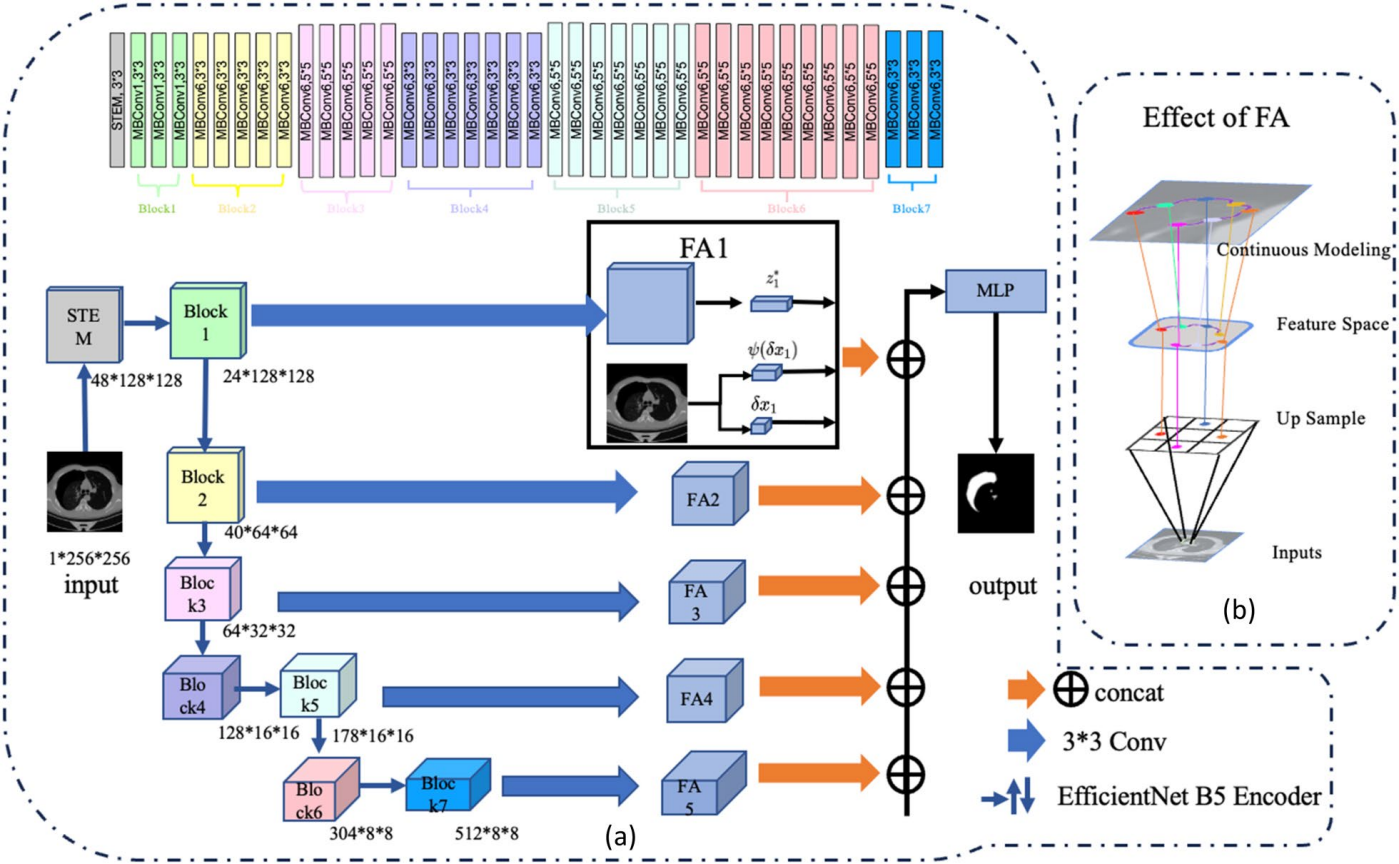
The pipeline of our proposed EFA-Net is illustrated in this section as shown in (**a**). The size of input image is 256 * 256, and model adopts an encoder-decoder architecture, with the left portion of the figure representing the encoder that employs EfficientNet-B5. The overall structure of EfficientNet-B5 is depicted in the upper middle of the image, showcasing the gray-shaded STEM, the green-colored Block1, the yellow-hued Block2, and continuing through to Block7. Following this, the output from each layer of the encoder undergoes convolution to derive the corresponding layer within the decoder. Inside each layer of the decoder, we view the features in feature maps as latent codes evenly distributed in 2D space. Given a query coordinate $x_{q}$, and the nearest latent codes is $\left\{ {z_{i}^{*} } \right\}_{i = 1}^{5}$ for each feature in feature maps $i$ and use $x_{i}^{*}$ to denote the coordinate of $z_{i}^{*}$. We then concatenate these latent codes $\left\{ {z_{i}^{*} } \right\}_{i = 1}^{5}$ and relative coordinate $\left\{ {\delta x_{i} = \left( {x_{q} - x_{i}^{*} } \right)} \right\}_{i = 1}^{5}$, and pass the concatenated vector into an MLP that directly predicts the segmentation label of point $x_{q}$. (**b**) Unlike methods such as linear interpolation, our FA decoder builds continuous feature maps, which can be generalized to arbitrary resolutions and retain finer details.

EfficientNetB5, depicted at the top of Fig. 3, was selected in one study due to its balanced trade-off between accuracy and training cost. The network consists of the following components: Stem Layers: These are the initial layers of the network responsible for preliminary feature extraction. Seven Primary Building Blocks of MBConv: These building blocks form the core of EfficientNetB5, utilizing Mobile Inverted Bottleneck Convolution (MBConv) for feature optimization and compression. The feature map resolution is progressively reduced five times, from 256 × 256 to 8 × 8 pixels, following the stem layers and blocks 2, 3, 4, and 6, respectively. This design helps to capture different aspects of the image at various scales. Through this structure, EfficientNetB5 offers an effective way to balance depth and width, reducing the number of parameters while maintaining high accuracy. Its balanced characteristics make it an ideal choice for various image segmentation tasks, including our specific application for pneumothorax segmentation.

**Figure 4:**
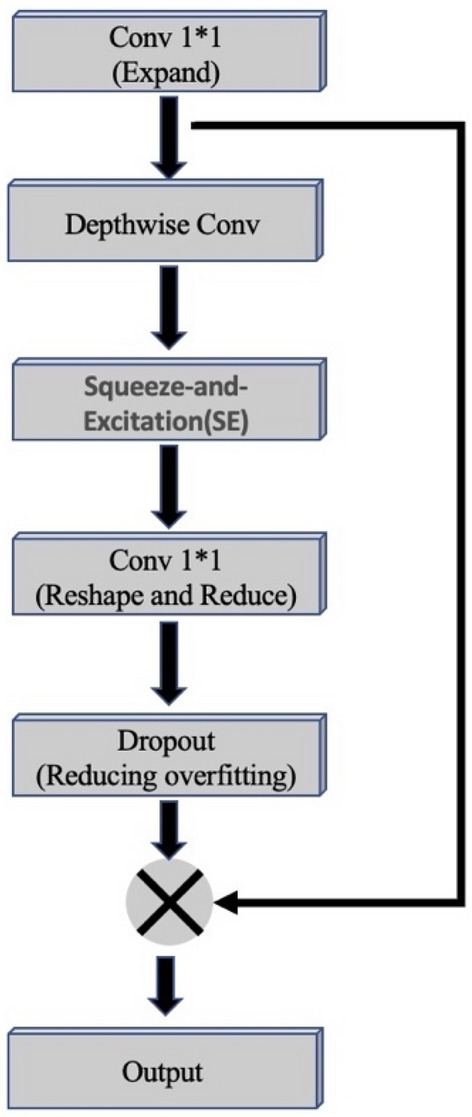
MBConv: Core building block of EfficientNet.

EfficientNet’s core building block is the mobile inverted bottleneck convolution (MBConv), which employs squeeze and excitation optimization, as illustrated in Fig. 4. The network can be scaled in three dimensions: width, depth, and input image resolution. Compound scaling of these dimensions can lead to significant improvements in accuracy. EfficientNet provides seven distinct versions, ranging from B0 to B7, each with increased depth, width, resolution, and model size, resulting in enhanced accuracy.

### Feature aligned function

Encoder-decoder architectures are commonly employed, irrespective of the complexity of the network layer combinations. In the task of pneumothorax segmentation, the objective is to map an RGB image $X \in {\mathbb{R}}^{{3{*}H{*}W}}$ or grayscale map $X \in {\mathbb{R}}^{{1{*}H{*}W}}$ to a semantic feature map $Y \in {\mathbb{R}}^{{1{*}H{*}W}}$. Here, H and W represent the height and width of the input image, respectively, and 2 denotes the number of classes. The encoder partially extracts features at various levels from the image through downsampling, while the decoder employs an upsampling module to restore the original image size. In a fully convolutional network (FCN), deeper network layers yield more fundamental features such as contours, edges, textures, and shapes of pneumothorax regions of interest (ROIs). However, less semantic feature information, such as ROI size and overall features, is obtained. State-of-the-art methods propose aggregating features from different levels to capture both local details and high-level semantic information. Following the UNet setting, different levels of features $F_{i} \in {\mathbb{R}}^{{C_{i} {*}H_{i} {*}W_{i} }}$ are extracted from various network stages, where i is the network stage number.

Decoder Function: The Feature Alignment Function’s decoder aims to define continuous feature maps (i.e., feature fields) that can be decoded at any coordinates, allowing for alignment in a continuous field without the need for up-sampling. We define Continuous Feature Fields ($D$): these are the feature maps that are continuous across coordinates. They are derived from the discrete feature map using the function $f_{\theta }$, Nearest Latent Code ($z$): this is a key concept in our decoder, where $z$ refers to the nearest latent code from the query coordinate $x_{q}$. It represents the most relevant feature at a specific location. Coordinate of Latent Code ($x$): this is the coordinate of the latent code $z$ signifying its position within the feature map.

1 $$D\left( {x_{q} } \right) = f_{\theta } \left( {z ,x_{q} - x } \right)$$

Feature Alignment and Position Encoding: Recognizing that neural networks may lack sensitivity to high-frequency signals, we employ the position encoding function $\psi \left( x \right)$ designed to encode spatial relationships between coordinates. This is achieved by applying the function $\psi$ to the relative coordinates $x_{q} - x^{*}$ as defined in the following:

2 $$D\left( {x_{q} } \right) = f_{\theta } \left( {z ,\psi \left( {x_{q} - x^{*} } \right),x_{q} - x^{*} } \right)$$

Here, $z$ represents the nearest latent code from $x_{q}$, and $x_{q} - x^{*}$ represents the relative coordinates between the query coordinate $x_{q}$, and the corresponding latent code coordinate $x^{*}$. By using $\psi \left( {x_{q} - x^{*} } \right)$, we transform these relative coordinates into a form that enhances the model’s ability to capture complex spatial dependencies.

Where the relative coordinates, along with their position encodings, are defined as:

3 $$\psi \left( x \right) = \left( {sin\left( {\omega_{1} x} \right),cos\left( {\omega_{1} x} \right), \ldots ,sin\left( {\omega_{L} x} \right),cos\left( {\omega_{L} x} \right)} \right)$$

The frequency $\omega_{l}$ is initially set as $\omega_{l} = 2e^{l} ,l \in \left\{ {1, \ldots ,L} \right\}$.This encoding strategy contributes to the robust handling of spatial relationships within the image.

To summarize the feature alignment function definition, we transform each feature map at various levels into a continuous feature map. This transformation allows us to access and align features at any coordinates, capturing both local details and high-level semantic information. As an example, we use $\left\{ {F_{i} } \right\}_{i = 1}^{5}$ (see Fig. 3). We use Feature Alignment function (FA) that directly generates a continuous feature map $D$ over multi-level discrete feature maps with different resolutions.

4 $$\begin{array}{*{20}r} \hfill {D\left( {x_{q} } \right)} & \hfill { = f_{\theta } \left( {\left\{ {z_{i}^{*} } \right\}_{i = 1}^{5} ,\left\{ {\psi_{i} \left( {\delta x_{i} } \right),\delta x_{i} } \right\}_{i = 1}^{5} } \right)} \\ \hfill {} & \hfill {} \\ \end{array}$$

5 $$\delta x_{i} = x_{q} - x_{i}^{*}$$

where $i$ denotes the index of the feature level, $z_{i}^{*}$ is the nearest latent code from $x_{q}$ at level $i$, and $z_{i}^{*}$. We implement $f_{\theta }$ as concatenating all its input vectors and passing them through a multilayer perceptron (MLP).

In summary, our proposed method utilizes the encoder-decoder architecture, with an emphasis on feature alignment for improved pneumothorax segmentation. By incorporating continuous feature maps at various levels, we can access and align features at any coordinates, capturing both local details and high-level semantic information. The integration of position coding further enhances the model’s ability to handle complex relationships between feature maps and spatial information. This approach paves the way for more advanced and accurate pneumothorax segmentation techniques in medical imaging applications.

### Ethics approval

The ethical aspect of this study was reviewed and approved by the Ethics Committee of Jiading District Central Hospital affiliated with Shanghai Health Medical College. All research methods were conducted in strict accordance with relevant guidlines and regulations. We hereby confirm that informed consent was obtained from all subjects and/or their legal guardians who provided data.

## Experiments

### Implementation details

The networks experimented in the Different Encoder and UNet Decoder sessions were implemented using the PyTorch framework and ten commonly used networks in the field of medical image segmentation. All networks were trained on an NVIDIA GeForce RTX-3090 (24 GB) GPU with 80 epochs and a batch size of 80, while in EFA-Net Ablation the batch size was set to 16. All training procedures used cross-entropy loss function and Adam optimizer. The learning rate was set to 0.001 during the whole training process.In the training process, we unify all dicom files to adjust the window width and window center to 1500, 600, then use the transformer of torchvision to adjust the image from 512*512 to 256*256 and then start training.

### Evaluation metrics

The confusion matrix is a statistical representation of network classification results. The confusion matrix consists of four regions of network prediction masks^24^: true positive (TP), true negative (TN), false positive (FP), and false negative (FN), as shown in Table 1. We employed five evaluation metrics, including accuracy (Acc) Dice coefficient (Dice), intersection over union (IoU), sensitivity (Sen), and specificity (Spec), to quantitatively evaluate the performance of the proposed method. The formal definitions are as follows:

6 $$Acc = \frac{TP + TN}{{TP + FP + TN + FN}}$$

7 $$Dice = \frac{2TP}{{2TP + FP + FN}}$$

8 $$IoU = \frac{TP}{{TP + FP + FN}}$$

9 $$Sen = \frac{TP}{{TP + FN}}$$

**Table 1:** Evaluation metrics.

Predicted mask/ground truth	Positive	Negative
Positive	TP	FP
Negative	FN	TN

### Ablation study

#### Different encoder and UNet decoder

Initially, we conducted ablation experiments to investigate the performance of nine commonly used medical image segmentation networks and the performance of Unet as an encoder combined with Unet’s decoder on our test set. The IoU and Dice scores obtained from the experiments were used as metrics to evaluate the performance of the models. Additionally, we recorded the number of parameters for each model, representing the model size. The results are shown in Tables 2 and Table 3. We found that EfficientNet as an encoder achieved significantly higher improvements in pneumothorax segmentation tasks compared to Unet’s original decoder.

**Table 2:** The ablation experiment results of module with nine different encoder and U-Net decoder on our dataset.

Methods	Accuracy (%)	IOU (%)	Dice (%)
Unet	99.77	73.66	83.46
se_resnext + Unet	99.72	70.82	81.78
res2net + Unet	99.73	70.52	81.23
gernet + Unet	99.72	70.30	81.43
resnext + Unet	99.70	68.72	79.74
resnest + Unet	99.76	72.28	82.45
se_resnet + Unet	99.76	72.13	82.26
regnetx + Unet	99.76	73.65	83.75
densenet + Unet	99.70	71.61	82.90
effientnet + Unet	99.80	77.98	86.88

**Table 3:** The FLOPs and Parameters of nine different encoder and U-Net decoder on our dataset, the results are calculated with a 1 × 1 × 256 × 256 input image.

Methods	FLOPs (G)	Pram (M)
Unet	7.76	24.43
se_resnext + Unet	7.74	24.89
res2net + Unet	10.84	32.66
gernet + Unet	8.48	27.37
resnext + Unet	15.74	51.14
resnest + Unet	10.10	24.43
se_resnet + Unet	10.32	35.05
regnetx + Unet	3.60	8.73
densenet + Unet	9.63	21.70
effientnet + Unet	2.92	3.04

When modifying the Unet model, we used nine common medical image segmentation models as the encoder to extract features from the input image. We compared the models’ feature extraction capabilities, and the final feature map was input into the original Unet decoder. The results are shown in Table 2. We found that EfficientNet not only significantly improved segmentation results but also had the fewest parameters. Therefore, we selected EfficientNet as the encoder component for our model.

#### EFA-Net ablation

To evaluate the performance of EFA-Net on pneumothorax CT segmentation task, we conducted ablation experiments. We chose Unet as the baseline model and compared it with the following four models: 1. UNet 2. Decoder is Unet, Encoder is EfficientNet 3. Encoder is Unet, Decoder is FA 4. Our work (Encoder part uses EfficientNet, Decoder part uses Feature Alignment Function (FA)). We kept all the models’ training data, hyperparameters, evaluation metrics, etc. the same to fairly compare their differences. We used Accuracy, IoU, Dice coefficient, as the evaluation metric, which can measure the degree of overlap between the segmentation results and the ground truth annotations. As shown in Table 4, we compared the four models on Dice coefficient. From the results, we can see that UNet itself performed worst on Dice coefficient, indicating that it could not handle pneumothorax CT segmentation problem well. Decoder is Unet, Encoder is EfficientNet and Encoder is Unet, Decoder is FA two models had some improvement compared to UNet but still worse than Our work (Encoder part uses EfficientNet, Decoder part uses Feature Alignment Function (FA)). This shows that both EfficientNet and FA parts have important roles in improving model performance. In particular, we found that FA could effectively align the feature representations between Encoder and Decoder and had adaptability and robustness.

**Table 4:** The ablation experiment results of module with nine different encoder and U-Net decoder on our dataset.

Methods	Accuracy (%)	IOU (%)	Dice (%)
Unet	99.77	73.66	83.46
EfficientNet + Unet	99.80	77.98	86.88
Unet + FA	99.82	79.75	86.90
EfficientNet + FA	99.83	81.80	90.03

### Performance and flops comparison of different methods

We validate our method by comparing it with six state-of-the-art methods, including UNet, UNet++, FPN^25^, LinkNet, TransUNet^26^ and DeepLabv3+. For a fair comparison, all methods are reproduced with the original code implementation given in their paper. In addition, the training environment and data preprocessing methods are ensured to be exactly the same. Table 5 reports the segmentation results on CT pneumothorax dataset. The remarkable performance of UNet++ and TransUNet also underscores the advances in deep learning-based segmentation methods, which contribute to the development of improved tools for pneumothorax detection and treatment.The parameters and FLOPs of each method are reported in Table 6. To further evaluate the efficiency of our proposed EFA method, we compared it with the other six state-of-the-art methods in terms of floating-point operations per second (FLOPs) and the number of parameters. The results presented in this section were calculated using a 1 × 1 × 256 × 256 input image for all models.

**Table 5:** The results of different methods on our dataset.

Methods	Accuracy (%)	IOU (%)	Dice (%)	SE (%)
Unet	99.77	73.66	83.46	81.56
UNet++	99.59	81.04	89.08	87.03
FPN	99.60	78.98	88.20	84.88
TransUnet	99.52	80.31	88.98	86.61
LinkNet	99.60	77.48	87.24	83.01
DeepLabV3+	99.62	80.07	88.84	85.48
EFA (our method)	99.83	81.80	90.03	88.94

**Table 6:** The FLOPs amd Parameters of different model, the results are calculated with a 1 × 1 × 256 × 256 input image.

Methods	FLOPs (G)	Pram (M)
Unet	7.76	24.43
UNet++	18.36	26.07
FPN	6.77	23.15
TransUnet	32.43	66.80
LinkNet	5.37	21.77
DeepLabV3+	7.82	22.43
EFA (our method)	1.55	0.43

Our EFA method demonstrated superior efficiency, achieving the lowest FLOPs (1.55 G) and the smallest number of parameters (0.43 M) among all the compared methods. In contrast, TransUNet exhibited the highest FLOPs (32.43 G) and the largest number of parameters (66.80 M), reflecting its more complex and computationally demanding architecture. Other methods, including UNet, UNet++, FPN, LinkNet, and DeepLabV3+, showed varying degrees of efficiency, with FLOPs ranging from 5.37 to 18.36 G and parameters ranging from 21.77 to 26.07 M.

The remarkable efficiency of our EFA method, in addition to its superior segmentation performance, highlights its potential for real-world applications in clinical settings where computational resources and time are often limited.

### Visualization of segmentation results

**Figure 5:**
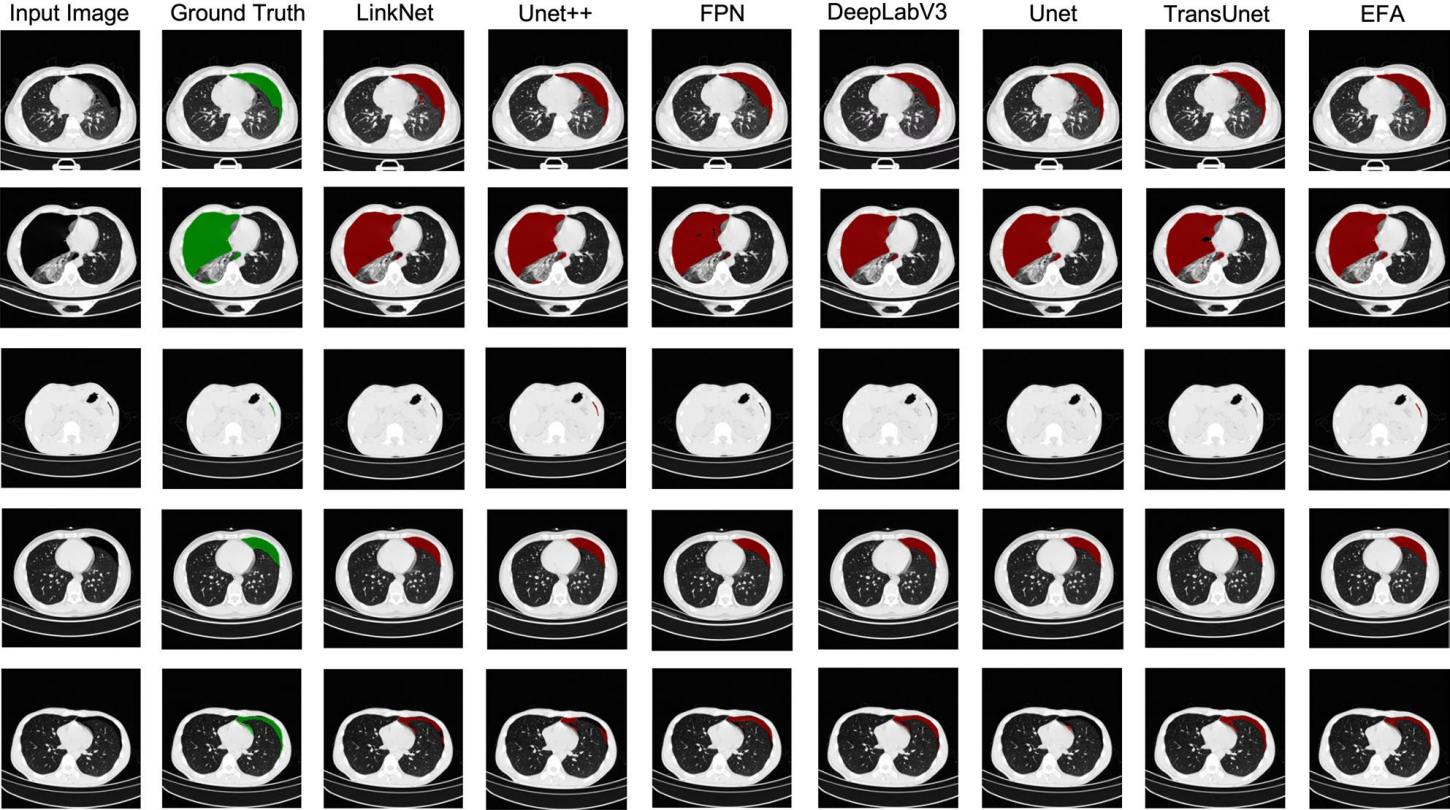
This image presents the segmentation results of randomly selected visualizations within the dataset. The first column displays the original image, the second column represents the Ground Truth, followed by different methods, and the final column shows our EFA-Net.

Figure 5 shows the segmentation results visualized in our dataset. The first column shows the original image, the second column represents the ground truth, then the different method columns, and the last column is our EFA-Net. The images in the first and fourth rows are relatively simple examples, and satisfactory segmentation results were achieved by almost all methods. However the second, third and fifth rows describe more challenging cases involving large target regions, very small ROI regions with irregularly shaped lesion regions. In the large region ROI in the second row each method can roughly identify the contours, in the small ROI in the third row only our method with Unet++ identifies the pneumothorax condition of the patient. To our surprise, the segmentation result of Unet++ is almost perfect in the first four rows, but in the irregular ROI in the last row there is an obvious case of ROI misidentification. Collectively, each method has ROI regions that are unique and good at segmentation, and EFA shows balanced and closest segmentation results to Ground Truth in each task.

## Conclusions

In this paper, we present EFA-Net, an innovative medical image segmentation network specifically designed for pneumothorax CT segmentation. EFA-Net incorporates EfficientNet as an encoder to extract features and a Feature Alignment (FA) module as a decoder to align feature maps of different sizes. Our method outperforms six state-of-the-art networks in segmentation performance, while exhibiting a lower number of parameters and FLOPs. Specifically, EFA-Net achieves a Dice coefficient of 90.03%, an IOU of 81.80%, and a sensitivity of 88.94% on our dataset. Notably, the network attains significantly lower FLOPs (1.549G) and parameters (0.432M), which in theory leads to better robustness and facilitates easier deployment when applied^27^.

Despite its advantages, there are still some limitations to our method, such as occasional missegmentation when the pixel intensity of the mass is close to the background and a dependency on manually labeled samples for training and the proposed EFA-Net was only tested on the pneumothorax CT dataset, and its generalizability to other medical image segmentation tasks remains to be investigated.

Recognizing EFA-Net’s potential for future advancements in medical image segmentation, we highlight semantic seg-mentation as the downstream deep learning task. Upon achieving a high level of accuracy in segmentation, EFA-Net can provide valuable insights, such as accurate segmented masks for pneumothorax species classification^28^, and enable the auto-matic calculation of a patient’s lung compression ratio end-to-end. This capability offers robust evidence to support clinical decision-making, including determining whether a patient requires a puncture surgery. As part of our future work, we aim to integrate these valuable downstream applications into the existing framework, ultimately enhancing EFA-Net’s utility for clinicians and patients in real-world diagnostic scenarios.

To address the limitations and extend EFA-Net’s applicability to other medical image segmentation tasks, we propose several research directions. One possible approach to overcome the missegmentation issue is to investigate the incorporation of additional context-aware features, such as attention mechanisms^29^ or multi-scale feature fusion^30^. These techniques can potentially help the model better differentiate between masses and background, leading to more accurate segmentation.

Another challenge is the reliance on manually labeled samples for training. To mitigate this, we suggest exploring semi-supervised or unsupervised learning methods for studying pneumothorax and other chest diseases in combination^31^. Leveraging a mix of labeled and unlabeled data can reduce the dependency on manual annotations. Transfer learning could also be considered as an alternative to improve generalizability^32^. By training the model on related medical image segmentation tasks, it might be possible to develop a more versatile medical model that can be fine-tuned for various applications. Moreover, incorporating domain adaptation techniques could be valuable in addressing dataset bias and improving the model’s performance on different medical imaging modalities^33^.

In conclusion, the EFA-Net proposed in this paper demonstrates promising results in pneumothorax CT segmentation, outperforming several state-of-the-art methods in terms of accuracy and efficiency. Despite its limitations, EFA-Net holds great potential for future advancements in medical image segmentation. As part of our future work, we will address the identified limitations and explore the integration of valuable downstream applications, aiming to enhance EFA-Net’s utility for clinicians and patients in real-world diagnostic scenarios.

## Data Availability

The data that support the findings of this study are available from the Jiading District Central Hospital, affiliated with Shanghai University of Medicine and Health Sciences, Shanghai, China. However, restrictions apply to the availability of these data, which were used under license for the current study, and so are not publicly available. Data are, however, available from the corresponding author upon reasonable request and with permission of the Jiading District Central Hospital, affiliated with Shanghai University of Medicine and Health Sciences, Shanghai, China. For data requests, please contact the corresponding author via email at robie0510@hotmail.com.
